# Review of Konjac Glucomannan Structure, Properties, Gelation Mechanism, and Application in Medical Biology

**DOI:** 10.3390/polym15081852

**Published:** 2023-04-12

**Authors:** Yilan Sun, Xiaowei Xu, Qinhua Zhang, Di Zhang, Xiaoyu Xie, Hanlin Zhou, Zhenzhen Wu, Renyi Liu, Jie Pang

**Affiliations:** 1 Center for Agroforestry Mega Data Science, Haixia Institute of Science and Technology, Fujian Agriculture and Forestry University, Fuzhou 350002, China; 2 College of Life Sciences, Fujian Agriculture and Forestry University, Fuzhou 350002, China; 3 College of Food Sciences, Fujian Agriculture and Forestry University, Fuzhou 350002, China

**Keywords:** konjac glucomannan, structure, property, gelation, medical biology

## Abstract

Konjac glucomannan (KGM) is a naturally occurring macromolecular polysaccharide that exhibits remarkable film–forming and gel–forming properties, and a high degree of biocompatibility and biodegradability. The helical structure of KGM is maintained by the acetyl group, which plays a crucial role in preserving its structural integrity. Various degradation methods, including the topological structure, can enhance the stability of KGM and improve its biological activity. Recent research has focused on modifying KGM to enhance its properties, utilizing multi–scale simulation, mechanical experiments, and biosensor research. This review presents a comprehensive overview of the structure and properties of KGM, recent advancements in non–alkali thermally irreversible gel research, and its applications in biomedical materials and related areas of research. Additionally, this review outlines prospects for future KGM research, providing valuable research ideas for follow–up experiments.

## 1. Introduction

Konjac glucomannan (KGM) is a natural macromolecular polysaccharide extracted from the konjac plant, which belongs to the Araceae plant family and is a perennial herb of the monocotyledonous class [1,2]. The molecular structure of KGM consists of β–glucose and β–mannose, with a small amount of acetyl groups, and it has a chain–like structure with a state of random curl [3]. The conformation of the single–chain of KGM is affected by dihedral angle energy, electrostatic interaction in vacuum, and hydrogen bonding in aqueous solutions [4]. KGM exhibits a high viscosity, solubility, and swelling property, a good film–forming property, and a good gel property in aqueous solutions [5]. The solubility of KGM in water is attributed to the presence of 5% to 10% acetyl substituent residues at mannose on the main chain of glucomannan [6]. Molecular dynamics simulation has shown that acetyl groups play a crucial role in maintaining the irregular semi–flexible helical chain conformation of KGM in aqueous solutions at room temperature, with the helical mode depending on its degree of polymerization [7].

The gelation mechanism of KGM involves osmotic transition, in which deacetylated KGM chains gather and produce nuclei, leading to gel formation [8]. Owing to its good biocompatibility, non–toxicity, gel property, and bio–friendliness, KGM has been widely used in the food industry and in medical biology [8]. As a soluble natural biological polysaccharide, KGM is considered as a preferred bio–based material [9]. Furthermore, KGM has been applied in biomedical materials, such as bio–matrix composites, which have made significant advancements in targeted drug delivery, the sustained release of drugs, and wound dressing [10].

In this review, we summarize the structure, properties, and gelation mechanism of KGM, as well as its application in biomedical materials.

## 2. Structure of KGM

### 2.1. Helical and Topological Structures of KGM

#### 2.1.1. Factors Affecting the Formation of the Helical Structure of KGM

The helical structure is an essential component in the construction of organisms, energy storage, and information transmission. In the study of KGM, researchers have discovered that the degree of polymerization, acetyl group, and temperature significantly affect the formation and stability of its helical structure [11,12].

The use of molecular dynamics simulation provided insight into the conformation of KGM at different degrees of polymerization, revealing an irregular helical structure with a more complex conformation as the degree of polymerization increased. The role of the acetyl group in maintaining the stability of the KGM structure was also explored, with the stable conformation of KGM containing an acetyl group being an extended helical structure. This stable conformation was found to be consistently maintained in dynamic simulation. However, KGM without an acetyl group was found to be in a random group state in vacuum, suggesting the critical role played by acetyl groups in the formation of the helical structure of KGM [7]. Furthermore, the influence of temperature on the helical structure of KGM was also investigated, with temperature having a reversible damage effect on the helical structure. The helical structure completely disappears at 341K, and the molecular chain presents a random mass structure. With decreasing temperature, the helical structure partially recovers [13]. The findings also suggest potential applications for KGM in the development of materials with tailored helical structures. However, further research is required to fully understand the complex mechanisms underlying the formation and stability of KGM’s helical structure.

#### 2.1.2. Active Fragments of KGM

KGM is a high–molecular–weight polysaccharide with a molecular weight range of 200–2000 kDa. While the high molecular weight of KGM provides good biocompatibility and degradability, it also limits its application scope. The biological activity of KGM varies significantly with different molecular weights [14], with a lower molecular weight indicating stronger activity. Therefore, exploring the degradation method of KGM is crucial [15]. Undoubtedly, the past several years have seen considerable progress in degrading KGM, such as enzymatic hydrolysis [16], acid hydrolysis, and physical hydrolysis [17,18]. However, enzymatic hydrolysis not only requires strict reaction conditions, but it is also hard to remove residual enzymes, which can be costly [19]. Acid hydrolysis often takes a long time to obtain the degraded products, which can affect the output and threaten the environment, causing permanent damage [20,21].

Previous studies have shown that degradation by γ–irradiation with suitable dosages may be an effective way to obtain degraded KGM with good dispersibility and without altering its basic chemical composition [22]. Jian et al. [23] obtained degraded KGM via convection irradiation at 100 kGy, which had good dispersion and specific physicochemical properties. They studied the protective effect of 100 kGy–KGM on oxidative damage caused by hydrogen peroxide in LO_2_ cells. The results showed that pretreatment with 100 kGy–KGM improved the survival rate of LO_2_ cells (human hepatic cell), hydroperoxidegutathione peroxidase, and catalase, while decreasing lactic dehydrogenase, malondialdehyde, and reactive oxygen species. It has been shown that 100 kGy–KGM has a good degree of antioxidant protection against oxidative damage, as well as good dispersity, making it suitable for use as an antioxidant. This study showed that γ–irradiation was an effective method to obtain suitably degraded KGM without changing the chemical groups of KGM.

Synergetic degradation uses two or more kinds of degradation techniques together to improve degradation. Synergetic degradation is more efficient than physical, chemical, or biological degradation alone, so it has been widely applied in the study of polysaccharide degradation. KGM was efficiently degraded by γ–ray irradiation in the presence of hydrogen peroxide through a synergetic effect [21,24]. This synergetic effect assisted cleavage of the β–1,4–glycosidic bond of KGM, promoting chain scission and the formation of carbonyl groups. The mechanisms of degradation via irradiation in the presence and absence of hydrogen peroxide differ significantly. Pan et al. [20] demonstrated that the crystallinity of KGM decreased when a KGM sample was irradiated with 50 kGy co–rays in the presence and absence of hydrogen peroxide. The synergistic effect of radiation and hydrogen peroxide was also found in the structural characterization of KGM. While the main chain of KGM did not change significantly after degradation, the physical characteristics of KGM were noticeably altered, potentially enhancing its application prospects.

Further efforts have been made to combine chemical and physical hydrolysis to degrade KGM, but inevitable shortcomings still exist. This is because they need a relatively long period to obtain the qualified degraded products. For instance, Jin et al. [25] combined γ–ray irradiation at a 6.0 kGy dose with ethanol to prepare degraded KGM, which takes several hours. Similarly, the alkaline–thermal degradation method was employed to hydrolyze KGM, which is still time–consuming [26]. A new strategy was reported to degrade KGM using a laser assisted with hydrogen peroxide; hydrogen peroxide was added to KGM solutions, as previous research has illustrated its excellent synergetic effect [20]. Lin et al. [27] degraded KGM using a laser with 10 W power, and the laser–degraded KGM (LDK) was characterized and analyzed. The viscosity and average molecular weight of LDK decreased, and the glycosidic bond of LDK was broken during laser irradiation. After degradation, the thermal stability of KGM improved. Moreover, LDK had a higher antioxidant activity than KGM, as demonstrated by the free radical scavenging activity of 1,1–diphenyl–2–picrylhrazyl. Overall, this strategy can provide a simple, time–saving, and cost–effective method for degrading KGM in an environmental–friendly way, and expand on the applications of KGM.

#### 2.1.3. Topology–Based Stabilization Mechanism of the KGM Chain

Topology is a mathematical field that studies the invariant properties of graphs after topological transformations. It enables us to build mathematical models from molecular structures and to predict the properties of chemical molecules. Thus, topology provides a microscopic basis for the synthetic chemistry of new molecules [12,28].

Hydrogen bond networks are a major unresolved problem in biology. As one of the main intermolecular interactions, hydrogen bond interaction has a significant impact on the properties of KGM [29]. When each internal element of a complex system is abstracted as a node, and the relationships between the elements are considered as links, a complex network is formed that reflects the topology. KGM is a stretching, rigid, half–flexible linear molecule with numerous groups on its surface. Under force, the groups can bind to some reactive molecules containing hydroxyl to form topological structures, which improves the stability of the active substance [30].

Topological analysis shows [31] that hydrogen bonding has a direct influence on the KGM system. There are many hydroxyl groups in the molecular chain of KGM, each of which may form hydrogen bonds. Epoxy atoms and glycoside oxygen atoms connect sugar rings with one or more water molecules. KGM is extremely hydrophilic, and each sugar ring can bind water molecules, showing a strong hydrogen bonding effect. The existence of the hydrogen bond plays an important role in its structural stability.

KGM nanogel microfibrils are usually prepared via electrospinning [32]. Fourier transform infrared spectroscopy (FT–IR) and field emission scanning electron microscopy are used to analyze the topological structure, and differential scanning calorimetry is used to detect the thermal stability of the structure. Chen et al. [32] showed that electrospinning increases the intermolecular hydrogen bond interaction and topological entanglement rate of KGM, and the nanogel microfibrils form a stable structure. These structures consist of topological networks of clustered nanofibers with lower porosity and higher density.

The binding conditions of KGM and epigallocatechin gallate (EGCG) molecules were calculated via thermodynamic analysis under electrostatic conditions [30]. Under macroscopic conditions, Hong et al. [30] synthesized KGM/EGCG nanofibers and obtained the most probable Mosaic model of KGM and EGCG at the microscopic level using Sybyl 8.1 molecular simulation software. Based on topological analysis and a series of features, EGCG was found, containing hydroxyl groups that can bind KGM molecules with a large number of hydrogen bonds, forming a “topologically protected structure of polyphenols embedded in KGM/EGCG” (embedded in the internal helix of KGM molecules), which improves the stability of polyphenols.

### 2.2. Chemical Structure and Modification of KGM

#### 2.2.1. Chemical Structure

The research on polysaccharide structure primarily focuses on the primary structure, including the composition and linkage order of monosaccharides, the molecular weight distribution, the ring type and conformation of monosaccharide residues, the α– or β–hybrid conformation of monosaccharide residues, the composition of branched chains, and their linkage sites. Additionally, substituted groups such as acetyl groups, sulfate groups, amino groups, and their substitution sites on the carbon skeleton of polysaccharides also contribute to the primary structure of polysaccharides.

Compared to other natural plant polysaccharides, the chemical structure of KGM is relatively simple. Numerous studies have shown that KGM is a heterosaccharide formed by β–1,4 glycosidic bonds of D–glucose and D–mannose with a molar ratio of 1:1.5 or 1:1.6, and its molecular formula is (C_6_H_10_O_5_)_n_ [2]. Short–chain branches may connect to mannose through β–1,3 glycosidic bonds at the C–3 position of the main chain of KGM. Moreover, acetyl groups are randomly distributed at the C–6 position of sugar residues, with approximately one acetyl group for every 19 sugar residues [33]. The acetyl group is a relatively important functional group on the molecular chain of KGM, significantly affecting the hydrophilicity and gelation of KGM. The heat treatment of KGM in an alkaline environment can remove acetyl groups and form a heat–irreversible gel.

Yui et al. [34] discovered that the glucomannan chain’s conformation displayed an extended two–fold helical structure on the X–ray diffraction (XRD) pattern, which was mainly formed by intermolecular O–3–O–5 hydrogen bonding and rotation on 0–6. Furthermore, the structure of KGM with different molar ratios of monosaccharides and varying acetyl group contents was analyzed using XRD. Based on the theory of network integration and minimum network entropy, KGM can form a left– and right–handed single–helical three–stage structure, where hydrogen bonding is the primary force in an aqueous solution [35]. However, the advanced structure of KGM is still not universally acknowledged. 

#### 2.2.2. Chemical Modification

Studies have been conducted to explore the modification of KGM in order to improve its performance. Chemical modification is one approach for altering the structure and properties of natural polymers, and various chemical modifications, including esterification [36,37], acylation [36,38,39], carboxymethylation [40], oxidation [41], and graft copolymerization [42], have been used. The abundant hydroxyl groups on the molecular chains of KGM provide active sites, which are significant for developing new functions of KGM and enhancing its application value.

KGM acylation modification is a type of esterification modification that produces new acylation products through the reaction between the hydroxyl groups on the molecular chain of KGM and acyl carbon chains. Wang et al. [43] investigated KGM acetylated polysaccharides with different degrees of substituents, and designed fibrous membranes based on acetylated KGM (Ace KGM) to modulate the activity of macrophages and to accelerate wound healing. Enzyme–linked immunosorbent assay results indicated that Ace KGM fiber membranes could enhance the expression of anti–inflammatory and pro–regeneration cytokines, and for Ace KGM, different degrees of substitution had a significant effect on the biological activity of the fibrous membrane. Ace KGM–based fiber membranes have potential applications as biological scaffolds for wound regeneration. 

The oxidation of glucomannan (OKGM) introduces carboxyl and carbon groups into KGM. This process reduces the molecular weight and generates new functional groups, thereby improving the sol’s stability. Yu et al. [44] used KGM oxide (DAK) as a macromolecular crosslinking agent to prepare gelatin–based drug hydrogels that could complete crosslinking and gelation within minutes. DAK promoted the formation of the gelatin network, and the DAK–treated gelatin hydrogels significantly slowed down the release of the model drug ketoprofen, with the release rate being adjustable by the ratio of DAK/gelatin and the pH of the buffer solution.

Carboxymethylation modification, which belongs to etherification modification, is a commonly used method for polysaccharide modification. It has been successfully applied to the modification of cellulose, starch, and other polysaccharides. The carboxymethylation of KGM also uses a large number of hydroxyl groups on the chain as the reaction site, and the carboxymethyl introduced in the reaction mainly locates at the C6 position of the sugar ring. Wu et al. [45] prepared a novel carboxymethyl KGM/chitosan (CMKGM/CS) nanogel with and without 1–ethyl 3–(3–dimethylaminopropyl)/n–hydroxy succinimide–induced crosslinking. The crosslinking did not change the size or morphology of the particles, but reduced the zeta potential of the nanogels compared to the original uncrosslinked nanogels. FT–IR confirmed the formation of amide bonds between CMKGM and CS, which greatly improved the stability of the nanogels in the gastrointestinal tract.

Graft copolymerization is another modification method that forms graft copolymers with special properties or new functions by linking polymer chain segments or functional side groups with different chemical structures to certain atoms of the polymer backbone. Xu et al. [46] synthesized KGM–grafted polyacrylamide at 25 °C under an N_2_ atmosphere using γ–radiation, and investigated the effects of absorbed radiation dose and monomer concentration on the grafting rate and water absorption rate. These results suggested that the γ–irradiation–induced graft copolymerization of KGM and acrylamide may hold promise for various applications where enhanced material properties are required. Further investigation could focus on optimizing the irradiation conditions and studying the copolymer’s behavior under different environmental conditions.

## 3. Formation Mechanism of a Non–Base–Irreversible Gel of KGM

### 3.1. Molecular Dynamics (MD) Simulation

Molecular simulation encompasses molecular dynamics, Monte Carlo, molecular docking, and other methods. Among these, MD is a powerful tool that can guide experimental studies [47]. The combination of MD simulation and experimental techniques represents an a priori means of solving biomedical problems. After years of development, molecular dynamics simulation technology has been applied in various fields of life science and has become one of the most important calculation methods connecting theory and experiment [48,49,50]. It is widely used in the food industry, biopharmaceuticals, materials, and other fields.

Hydrogen bonds are weak intermolecular or intramolecular interactions found widely in natural polymers, such as carbohydrates, amino acids, and proteins [51]. They have a significant influence on the structure and properties of natural polymers, such as crystal folding, molecular configuration, the stability of the complex, and the activity of biomacromolecules. When the number of hydrogen bonds in the system increases to a certain extent, the sol–gel phase transition occurs, and the hydrogen bond network appears. Therefore, the effect of adding different components to the structure of the KGM network was studied using molecular dynamics simulation [52].

Hydrogen bonds play an important role in maintaining the structural stability of KGM [29]. According to the molecular dynamics simulation results of Pang et al. [53], after acetyl group dissociation at pH = 11, more hydrogen bonds are formed, and the KGM structure is more stable. Under the same pH condition, KGM forms more hydrogen bonds at a temperature of 368K and has a more stable conformation. However, when urea is added to KGM solution, the number of hydrogen bonds is reduced, and the structure’s stability is decreased [29]. In conclusion, the change in hydrogen bonds affects the structural stability of KGM and ultimately leads to a change in gel properties [54].

The hydrogen bond network structure formed by the interaction between NH_2_–KGM–Zn and water was studied using molecular dynamics simulation [51]. Compared with da–KGM, the NH_2_–KGM–Zn complex increases the number of hydrogen bonds, improves the gel strength, and interacts more strongly with water. This provides a theoretical basis for the study of a KGM–irreversible gel using the NH_2_–KGM–Zn complex.

Molecular dynamics simulation showed that the gel performance of a composite gel is better than that of a single–component gel [54]. According to research on the effect of biomacromolecule mixing, it is often observed that the strength, elasticity, and stability of a mixed gel are enhanced compared to a single–component gel [55,56]. KGM has a synergistic effect with other polysaccharides or proteins under certain conditions. Molecular dynamics simulations are used to study the details of the interactions so that the interaction properties of mixed gels can be better understood. The stability of the KGM gel is predicted by potential energy, surface area, and hydrogen bond analysis by Wang et al. [57]. It is found that the structural stability of the mixed gel is higher than that of a single gel. Moreover, the energy changes of the gel system are simulated, and it is found that intramolecular and intermolecular hydrogen bonds can be formed in the composite gel, resulting in a gradual reduction of the conformation energy obtained by the KGM composite system and then close to stability [58].

The limitations of traditional research methods results in the slow development of research at the molecular level [58]. By making full use of computer–aided simulation and quantum mechanics, the efficiency and depth of research can be improved.

### 3.2. Properties of KGM Polysaccharide Gels

Polysaccharides derived from natural sources are widely used in biomedical applications due to their unique gelling properties [59]. Polysaccharide gels are found to be thermo–reversible, making the development of thermo–stable polysaccharide gels essential. KGM gels as polysaccharide gels can be divided into three types: thermally irreversible gels prepared with alkaline coagulant, thermally reversible gels prepared with boron, and synergistic reversible gels prepared with other polysaccharides or proteins.

**Figure 1:**
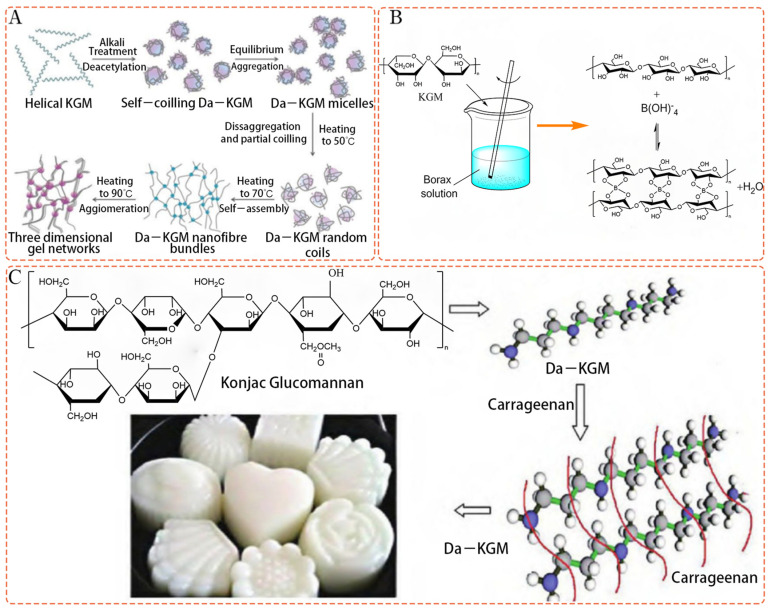
(**A**) Alkali–induced gelation mechanism of KGM (Adapted with permission from Ref. [6]. 2023, Elsevier); (**B**) Schematic diagram of the formation mechanism of KGM–borax hydrogel (Adapted with permission from Ref. [62]. 2023, Springer nature); (**C**) Schematic diagram of a gel of KGM with carrageenan (Adapted with permission from Ref. [65]. 2023, Elsevier).

KGM can form thermally irreversible gels in the presence of an alkali coagulant since alkali treatment removes acetyl groups and reduces water solubility, which increases absorbency and promotes wound healing [60]. KGM transits from random coil configurations to more stiff configurations accompanied by self–assembly to aggregate with one another and form junction zones. Thus, hydrogen bonding and hydrophobic interactions are the main forces of gel formation [6]. Additionally, KGM can also react with borate to form thermally reversible gels [61,62]. The gel network is formed by the crosslinking of borates and cis–diol hydroxy groups on KGM [63]. The mechanism of the interaction between KGM and boric acid has been studied using molecular simulation technology. According to Jian et al. [64], through computer simulation, it is found that the hydroxyl group of B (OH)^4−^ forms hydrogen bonds, with the hydroxyl group of C_6_ on KGM glucose and O_5_ on mannose, but the KGM molecular chain remains in a helical conformation, and borate ions are embedded between the KGM molecular chains. KGM is also capable of forming reversible complex gels with other polysaccharides or proteins, such as xanthan, carrageenan, and agarose [65]. Three types of gelation mechanisms for KGM gels are presented in Figure 1.

However, these three gels all have certain limitations. For example, more than 90% of KGM gels, prepared in the presence of an alkaline coagulant, exhibit a high syneresis rate, color absorption, and strong alkali taste. To remove the strong alkali taste, the gel is washed repeatedly with water or acid, which affects the quality of the product. According to Wang et al. [66], the acetyl content of the gels obtained at different electric treatment times decreased, showing that KGM was partially deacetylated during the gel formation, which also indicated that KGM was partially deacetylated under an AC electric field and KCl. For KGM–borax gels, the toxicity of boron ions seems to limit their practical use [67]. The toxicity of boron ions limits the practical use of KGM–borax gels [67], indicating that finding borax substitutes that can form complexes with the groups within KGM molecules is a feasible research direction. Gels prepared using a mixture of polysaccharides show poor thermal stability [68].

Therefore, studying the possible formation mechanism of gels can provide theoretical understanding and a new method for the preparation of new KGM gels.

### 3.3. Physical Parameters Regulate the Properties of Non–Alkali Irreversible Gelation

The gelation process of KGM is influenced by various factors, including temperature, ions, microwave, ultra–high static pressure (HHP), etc. [69]. Further research is needed to study the gel behavior and interaction mechanism of KGM with different ion types (e.g., Ca^2+^, Zn^2+^, Fe^3+^, etc.), concentration, and physical fields (e.g., ultrasonic wave, microwave, pulsed electric field, HHP, etc.). KGM can also form complex gels by interacting with other polysaccharides or proteins, such as xanthan gum, carrageenan, agarose, etc. The addition of metal ions to the gel and the application of an electric field can enhance the gel properties of KGM gels.

Studies have shown that the addition of metal ions to the complex gel enhances its gel strength (Table 1). Although the properties of a gel formed by κ–carrageenan (KC) and KGM can be improved, the thermal stability during heating treatment is still inadequate for practical applications [68]. However, the presence of metal cations (e.g., K^+^, Na^+^, Ca^2+^) at a certain amount can enhance the gel strength under storage conditions, with different performances [70]. Gellan gum (GG) is a linear anionic polymer that forms a transparent gel in the presence of cations, similar to the gelation of KC. Several studies have introduced GG and/or Ca^2+^ to improve the mechanical properties and thermal stability in the KC/KGM hybrid system [71]. The impact of Ca^2+^ and GG on the properties of the thermo–reversible gel system (KC/KGM) were investigated using two deformation test, rheology, micro–rheology, and SEM. The results showed that either GG or Ca^2+^ alone raised the Tgs and Tsg of KC/KGM gels, but did not prevent the KC/KGM gel from melting during the heating process. However, when appropriate amounts of GG and Ca^2+^ were added to the KC/KGM gel together, the KC/KGM gels were transformed into thermally irreversible gels during the heating process. This research enriched the types of gel that can be used in food and medical materials.

In addition to aiding in the formation of KGM gels, the application of electric fields can enhance their thermal stability. Wang et al. [72] successfully prepared KGM–tungsten gels at 0.3% KGM concentration in the presence of sodium tungstate under direct current (DC). The gels demonstrated high thermal stability and electrochemical reversibility. The rheological properties of the gels were investigated, and it was found that the storage and loss moduli increased with the addition of sodium tungstate, voltage, and processing time. However, the concentration of KGM had an inverse relationship with the moduli. The possible mechanism of gel formation was that WO_4_^2−^ was absorbed onto the KGM chain and crosslinked with the OH group at C–6 on KGM under DC, followed by the assembly of KGM molecules to the anode to form a 3D gel network.

KGM can also form a gel with the assistance of KCl under an alternating current (AC) electric field [66]. This method only requires a current and electric field strength, and KCl and KGM concentrations without the need for heating. The KGM–KCl electrogel demonstrated a high thermal stability, with a decomposition temperature of about 250 °C, and a reduced KGM crystallinity due to the adsorption of K^+^ and Cl^−^ ions. The formation of the KGM–KCl electrogel proceeded through a simpler and more controllable method compared to DC. However, the formation process takes longer and can break the KGM molecular chains, resulting in relatively smaller and thinner gels compared to DC.

Both the DC and AC electric fields can be used to prepare KGM gels with strong thermal stability, broadening their potential applications in food, materials, and other fields [64]. Compared to DC, AC has some advantages, such as the use of non–toxic KCl instead of toxic sodium tungstate, and a greater sensitivity to small changes in voltage. However, the KGM–KCl electrogel takes longer to form, and AC can break KGM molecular chains [73].

Overall, the application of electric fields to prepare KGM gels offers a promising avenue for the development of novel materials with unique properties. Further investigation is necessary to optimize the preparation method and to understand the underlying mechanisms of gel formation under electric fields.

**Table 1:** Reductions of metallic nanoparticles for multifarious applications using KGM.

Materials	Properties	References
KGM, silver nanoparticles	Improved mechanical performance, outstanding antibacterial efficacy, extended water retention period, and excellent water absorption capacity	[74]
KGM, gelatin (G), gold nanoparticles	Favorable mechanical and antibacterial characteristics, exceptional water absorption and retention capabilities	[75]
KGM, CS, AgNPs	Swelling capacity, mechanical properties, and biocompatibility	[76]

## 4. Application of the KGM Gel in Biomedical Materials

KGM has gained increasing attention in the field of medical materials due to its unique properties such as gelation, water retention, water solubility, and biocompatibility. For instance, KGM has been utilized to prepare highly stable nanosilver sol, which exhibits exceptional oxidation stability and biocompatibility. Furthermore, silver nanoparticles can be employed to develop antibacterial materials, such as antibacterial dressings containing silver ions, which have been shown to be highly effective in promoting wound healing while being safe and reliable. Additionally, KGM can serve as a carrier for exocrine or stem cells, enabling targeted drug delivery with excellent biocompatibility, stability, and safety, with anti–inflammatory and sustained–release properties. KGM has also been used to develop disposable syringes, which offer several advantages such as low carbon emissions, environmental friendliness, strong antibacterial properties, good printability and adhesion, and high toughness, while being resistant to rupture and the contamination of drugs. KGM has tremendous potential in the field of medical materials, making it a promising candidate for future research and development.

### 4.1. Wound Dressing Material

Skin regeneration is a pressing issue due to the high incidence of skin injuries. Protein–based synthetic skin scaffolds have been effective in this regard, but the development of skin–like extracellular matrix scaffolds that possess outstanding biocompatibilities, superior antibacterial properties, robust mechanical capabilities, and that are cost–effective remains a significant challenge [77]. KGM has been widely used as wound dressing materials (Table 2). KGM–based wound dressings can provide a moist environment that is conducive to wound healing, as well as effective antibacterial properties. Zhao [78] described an invention that relates to an antibacterial heal–promoting gel material used for preparing medical wound dressings with collagen protein, sodium alginate (SA), agar, KGM, and bacterial cellulose. Han et al. [79] provided a new medical material, and in particular, this was related to a high–expansibility medical polysaccharide–based material with KGM. Feng et al. [80] explored the physical crosslinking of silk fibroin (SF) and KGM to produce biocompatible protein/polysaccharide sponges with tunable mechanical properties for wound dressings. The sponges’ SF/KGM mixing ratio could be adjusted to create a uniform and interconnected pore structure. Increasing the KGM concentration improved the compressive strength, water absorption, and water retention ability of the porous sponges. The composite sponge displayed good biocompatibility, a moist environment, a compression modulus comparable to skin tissue, and a high capacity for water absorption.

In another study, Gomes et al. [81] employed CS and KGM as raw materials in a two–step casting process to create a new bilayer film. A hybrid film was also created to investigate the interaction of the two polymers at the interfacial region of the bilayer structure. SEM, FT–IR, and XRD analyses revealed that the bilayer film retained the physicochemical properties of each biopolymer compared to the mixture. Both polymers exhibited good thermal stability and miscibility, which may be due to the efficient hydrogen bonds between their polymer chains. The bilayer film demonstrated good biocompatibility, a low cytotoxicity, and mechanical and barrier properties suitable for wound dressings, as determined by tests on biological, mechanical, and water vapor transmission.

More investigations are required to better understand the underlying mechanisms of wound healing, and the interaction between KGM and other biomaterials. In addition, studies exploring the long–term biocompatibility, stability, and efficacy of KGM–based wound dressings are necessary. Further research can also focus on the development of KGM–based wound dressings that possess tailored mechanical and degradation properties to meet specific wound healing requirements.

### 4.2. Biological Scaffold

The use of biological scaffolds for bone regeneration has been extensively studied, with KGM emerging as a promising candidate due to its excellent cytocompatibility and osteointegration. Several studies have investigated the properties of KGM–based scaffolds and their ability to promote bone healing.

Zhao et al. [86] and Kanniyappan et al. [87] have reported on the use of KGM–based scaffolds in bone tissue engineering. Zhao et al. [86] incorporated KGM and ferric amine (DFO) bioactive coatings into porous hydroxyapatite scaffolds (HA) to enhance self–resorption and vascularization. The KGM covering induced HA/DFO/OKGM (HOD) self–resorption, and DFO stimulated damaged bone vascularization. The resulting bioactive–coated scaffold demonstrated excellent osteogenic differentiation and vascularization. Micro–computed tomography imaging revealed that HOD was degraded at a rate matching the growth of new bone, leading to efficient bone healing in rats.

Similarly, Kanniyappan et al. [87] created binary and ternary interpenetrating network (IPN) scaffolds using natural and synthetic polymers, including KGM and polyvinyl alcohol. The resulting scaffolds had a microporous structure with strong interconnectivity, and showed non–toxicity and osteointegration in vitro. The mechanical strength of the ternary scaffold was comparable to that of real bone. Gene expression analysis confirmed the scaffold’s ability to stimulate osteogenic differentiation.

Another invention presented a method for providing the repair of cartilage graft support with polysaccharide solution, including KGM [88]. Pan et al. [89] provided a preparation method and application of a cell–scaffold composite material for repairing cartilage injury by mixing KGM and sodium hyaluronate, and the cells and the scaffold were subjected to a co–culture method to obtain the cell–scaffold composite material. An invention provided by Chen et al. [90] related to a hydroxyapatite, sodium hyaluronate, and KGM porous scaffold composite material for bone defect restoration and a preparation method thereof, and it belongs to the field of biomedical materials.

These studies provide valuable insights into the potential use of KGM–based scaffolds in bone tissue engineering. The incorporation of bioactive coatings and the use of IPN scaffolds can enhance their ability to promote vascularization and osteogenic differentiation, leading to faster bone healing. However, further research is needed to optimize their properties and clinical applicability to overcome current limitations in biological scaffolds for critical size bone defects.

### 4.3. Drug Carriers

Natural polymers have been gaining attention as promising candidates for drug carriers due to their biocompatibility and biodegradability. KGM has been utilized in various forms such as nanoparticles, emulsions, tablets, and hydrogels for targeted drug delivery systems that respond to different physiological conditions (Table 3). Researchers have explored the potential of KGM in combination with other polymers for improved applications. For example, Xu et al. [91] developed concanavalin–insulin–KGM nanoparticles loaded with insulin for oral delivery in response to blood glucose levels, while Wang et al. [92] fabricated Ace KGM nanoparticle–loading curcumin for colon–targeted release targeting inflammatory macrophages. Inhaled therapy for tuberculosis has also been investigated using KGM microparticles prepared through spray drying techniques [93,94].

Despite its advantages, KGM systems have faced challenges such as pH sensitivity and water absorption behavior. Wang et al. [95] prepared a new EGCG–supported hydrogel using dopamine–carboxymethyl KGM and L–cysteine–carboxymethyl KGM as raw materials and Fe^3+^ as a crosslinking agent, demonstrating potential applications in controllable polysaccharide materials. In biomedical engineering, functional bio–inspired hydrogels with good release control capabilities are crucial. Wang et al. [96] developed a composite hydrogel using carboxymethyl KGM, gelatin, and tannic acid–functional nanohydroxyapatite, exhibiting good biodegradability and pH sensitivity. Wu et al. [45] created a crosslinked CMKGM/CS nanogel for improved stability and the sustained–release behavior of curcumin under simulated gastrointestinal environments.

Three–dimensional hydrogels have emerged as attractive drug delivery systems that can be formulated to provide controlled drug release profiles. However, pure κ–carrageenan hydrogels have a drug burst release behavior that greatly limits their applications. This work suggests that KGM has the potential to enhance the properties and drug release characteristics of soy protein isolate/κ–carrageenan (SPI/KC) composite hydrogels.

To improve the bioavailabilities of active substances and to reduce their toxic and side effects on the human body, natural biological macromolecules are used to load active substances and to control their release speed in different environments of the human body. Dao et al. [97] combined mesoporous silica loaded naringin gel spheres (MSNs) with KGM and SA to prepare pH–sensitive KS/MSN gel spheres. The KS/MSN composite gel spheres showed the best slow–release effects in a simulated small intestinal fluid environment, and the overall amount of NG released showed a slow increasing trend with the highest final release amount.

KGM hydrogel has favorable gel–forming abilities, but its insufficient swelling capacity and poor control release characteristics limit its application. Therefore, Wu et al. [98] used oxidized hyaluronic acid (OHA) to improve the performance of the KGM hydrogel. The effect of the OHA content on various gel properties was evaluated. The obtained hydrogel was pale yellow, smooth in surface, and had a favorable swelling capacity, which qualified the essential requirements for ideal drug delivery applications. The overall results suggest that the KGM/OHA hydrogel, loaded with EGCG, exhibited potential applications in controlled release.

The strong rigidity and brittleness of pure agarose (AG) hydrogels limit their practical applications. To address this, Yuan et al. [9] incorporated KGM to enhance the properties of the AG hydrogel. The in vitro drug release behaviors of the composite hydrogels were investigated under different conditions, using ciprofloxacin (CPFX) as a model drug. The results showed that the encapsulation efficiency, drug loading capacity, and sustained–release capacity of AG hydrogels were improved by the inclusion of KGM. These findings suggest that KGM has the potential to enhance the properties and drug release characteristics of AG hydrogels.

Overall, natural polymers such as KGM exhibit a great potential as carriers for targeted drug delivery systems, with improved biocompatibility, biodegradability, and release control capabilities. 

**Table 3:** KGM as drug carriers.

Matrix	Drug	References
KGM, GG	Dihydromyricetin	[99]
KGM, functionalized CCNT	5–fluorouracil	[3]
CMKGM KGM, CS	Curcumin	[45]
CKGM, G, tannic acid functional nanohydroxyapatite	EGCG	[96]
KGM	Isoniazid, rifabutin	[94]
KGM, concanavalin A	Oral insulin	[91]
KGM, SPI, κ–carrageenan	Glucose	[56]
KGM, OHA	EGCG	[98]
KGM, MSN, SA, NG	NG	[97]
KGM, AG	CPFX	[9]

### 4.4. Endodontic Treatments

Although tricalcium silicate (TCS)–based cement has shown great clinical success as a dental filling material, one of the major challenges encountered by TCS is its poor anti–washout property. To address this challenge, Wu et al. [100] developed a novel TCS/calcium formate (CF) cement that uses KGM solution as a liquid with excellent anti–washout ability for the first time. The addition of CF not only enhances the washout resistance of TCS, but also accelerates the hydration kinetics, resulting in a decreased setting time and a more compact microstructure. The researchers investigated the self–setting characteristics, anti–washout ability, setting time, compressive strength, porosity, injectability, and flowability of the cement. They also assessed the antibacterial property and cell cytocompatibility of TCS/CF. The results showed that KGM could enhance the washout resistance of TCS pastes while hindering the hydration reaction. CF can further increase the anti–washout ability of TCS cement and shorten its setting time from 420 to 174 min due to the accelerating effect of CF on the hydration kinetics of TCS. Compared to TCS pastes, TCS cement containing CF showed increased compressive strength, while the addition of CF decreased the injectability of pastes without an obvious effect on flowability. The antibacterial activity of TCS/CF against S. aureus increased with the amount of CF. Moreover, TCS/CF had good cell cytocompatibility. Therefore, TCS/CF with a good anti–washout ability might be an attractive candidate as a dental filling cement, and the addition of KGM can be further investigated with other materials. 

### 4.5. Three–Dimensional Printed Therapeutic Agents

Three–dimensional (3D) printing hydrogels offer a combination of advantages from both 3D printing and hydrogel technology. Hydrogels with 3D printing exhibit excellent physical and chemical properties, such as a high mechanical strength and excellent electrical conductivity [101].

Wang et al. [102] prepared composite hydrogels through a Schiff–base reaction between the aldehyde group of OKGM and the amino group of branched polyethyleneimine (PEI). The resulting OKGM/PEI composite hydrogel exhibited self–healing ability, pH sensitivity, and shear–thinning capability, which is suitable for 3D bioprinting. By adding carbon nanotubes (CNTs), the OKGM/PEI electroactive composite hydrogel was obtained, which had its rheological behavior and morphology characterized. The conductivities of OKGM/PEI electroactive composite hydrogels increased with increasing CNT content. With a lower addition of 2% CNTs, the conductivity of OK16P2–CNTs (a mixture sample of OKGM/PEI/CNTs) reached 10^−4^ S/cm, satisfying the needs of cells for micro–current stimulation to promote proliferation and differentiation. CNTs also improved the bio–printability of OKGM/PEI electroactive hydrogels. Therefore, the OK16P2–CNTs electroactive hydrogels have potential applications in tissue regeneration and could be employed as scaffolds for muscle and cardiac nerve tissue regeneration.

In cartilage tissue engineering, a three–dimensional (3D) bio–ink with favorable print–ability, strength, and biocompatibility is highly desirable. Qin et al. [103] innovatively developed methacrylated KGM (KGMMA) as a new bio–ink for 3D bioprinting. The KGM hydrogel could be directly printed into a temporally stable 3D structure at low concentration. After UV crosslinking, the KGMMA hydrogel formed a covalent crosslinking network with high strength, desired shearing, and swelling and degradation characteristics. The structure, swelling, and biodegradation behaviors, and the mechanical properties of the KGMMA hydrogel could be adjusted by modulating the degree of methacrylation (DM) of the KGMMA polymer. The shear–thinning characteristic of the KGMMA hydrogel provided excellent printability for cells, facilitating cell activity. Therefore, the biodegradable, injectable, and photo–crosslinkable KGMMA hydrogel holds promise for cartilage tissue engineering.

KGM exhibits unique properties, such as high viscosity, water–holding capacity, and easy gelatinization, which make it a promising material for bio–printing applications. However, only a limited number of studies have explored its 3D bio–printability, and further research is needed to fully assess its potential in this field [104].

Apart from its potential as a biomedical material, KGM has demonstrated efficacy in various pharmaceutical and clinical applications. Clinical trials have shown that KGM can positively impact weight management, blood glucose regulation, and lipid profile improvement [105,106]. Moreover, KGM has potential therapeutic benefits for patients with metabolic syndrome and diabetes [107,108]. As a dietary supplement, KGM has been used to lower cholesterol levels, while also serving as a food additive to enhance the texture and viscosity of food products [109,110]. Furthermore, KGM has been investigated for its potential as a drug delivery system, enabling the controlled release of drugs within the body [3,91]. The therapeutic potential of KGM appears promising, yet further research is necessary to gain a comprehensive understanding of its clinical applications.

## 5. Research Prospects of KGM in Medical Biology

### 5.1. Multiscale Simulation of the KGM Sol Mesoscopic System

The successful injection of macromolecular solutions, the atomized breakdown of macromolecules, and the high–speed transport of colloids all rely on the rheological characteristics of bio–macromolecular colloids under shear rates exceeding 103 s^−1^. However, analyzing the impact of the dynamic cluster structure on the rheological properties, and characterizing the rheological properties at high shear rates using standard equipment is challenging.

**Figure 2:**
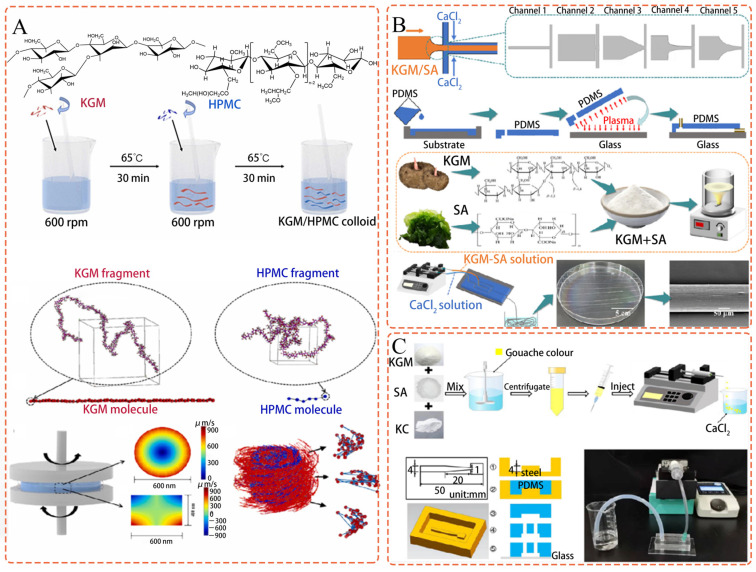
(**A**) Creating the KGM and HPMC composite colloid and displaying the multi–scale simulation method’s schematic (Adapted with permission from Ref. [111]. 2023, Elsevier); (**B**) Diagrammatic representation of the production of biofibers and microfluidic chips (Adapted with permission from Ref. [112]. 2023, Elsevier); (**C**) Spherical hydrogel and device preparation (Adapted with permission from Ref. [113]. 2023, Elsevier).

To address this challenge, Liu et al. [111] investigated the rheological characteristics of biomolecular colloids composed of KGM and hydroxypropyl methylcellulose (HPMC) using multi–scale simulations and tests. By combining molecular dynamics simulations with Brownian dynamics simulations, the authors reliably and successfully studied the dynamic behavior of long–lived nanoclusters. Notably, multi–scale simulations were used for the first time to predict cluster structure and rheological characteristics in strong shear flows, including polymer fracture and a decrease in storage modulus at high shear rates (104 to 105 s^−1^), which were only imperfectly measured using traditional instruments. Figure 2A shows the schematic of creating KGM and HPMC composite colloids, and displaying the multi–scale simulation method. This study sheds light on the rheological properties of bio–macromolecular colloids, providing a theoretical basis for their application in strong shear flows.

### 5.2. Multiscale Simulation of KGM Mesoscopic Clusters in Microfluidic Spinning

Micro– and nanofibers have been produced on a large scale through microfluidic spinning. However, the impact of chip channel geometry on the tensile properties of micro–nanofibers is not well understood. To address this, Xu et al. [112] examined the microstructures and tensile properties of fibers made from KGM and sodium alginate using a multi–scale numerical technique. Figure 2B shows a diagrammatic representation of the production of biofibers and microfluidic chips.

The authors used this technique to simulate the dynamic behaviors of macromolecular clusters in the flow field. They found that the tensile strength and strain of long fibers were 1.37 times and 1.5 times higher, respectively, than those of fibers produced under shear flow conditions with increased flow and tensile rates. The tensile strength of the long fibers was found to be 32.34 MPa and 18.72%, respectively. The different tensile qualities were primarily influenced by the microscopic shape of the flow modulation field.

Notably, the flow field, the dynamic behaviors of molecular clusters, the morphologies of the fibers, and the tensile characteristics were all significantly affected by the channel form. Overall, this study provides a new numerical approach to studying microfluidic spinning and expands our understanding of the factors that influence the production of micro– and nanofibers.

### 5.3. Mechanical Test Approach and Analysis of the KGM Gel during Deconstruction

Spherical hydrogels have a wide range of applications in the food, biological, and pharmaceutical industries, but accurately assessing their mechanical properties is crucial for their effective use. However, real–time quantitative assay techniques for soft spherical hydrogels are currently lacking. To address this, Zeng et al. [113] proposed a technique that combines numerical calculations and hydrodynamic loading tests. The preparation of a spherical hydrogel and device is shown in Figure 2C.

In their study, the authors used fluid forces to inject spherical hydrogels into constricted channels for hydrodynamic loading tests. Based on the experimental results, they calculated the hydrodynamic forces and the apparent modulus of the spherical hydrogel using computational hydrodynamics and finite element simulations. The effectiveness and accuracy of the hydrodynamic loading experiments and the numerical simulation (HD–NS) method were confirmed by evaluating the apparent moduli of composite spherical hydrogels made of KGM, sodium alginate, and carrageenan over a range of 8.93–35.75 kPa.

Next, the authors used the HD–NS method to calculate the apparent modulus and the deformation of the spherical hydrogels during deterioration. The results showed that the microstructures of the spherical hydrogels became looser, and the apparent modulus gradually decreased from 30.48 kPa to 9.14 kPa with a prolonged deterioration time or a decreased pH value of the solution. These findings provide a better understanding of the relationship between the degree of degradation, microstructure, and mechanical properties of spherical hydrogels during deterioration. 

This research has significant implications for the development and utilization of biodegradable spherical hydrogels with precisely controlled mechanical properties, as they can be used in the creation of synthetic blood cells and in sustained–release delivery systems for both injectable and consumable drugs. In summary, the proposed technique represents a valuable tool for accurately assessing the mechanical properties of soft spherical hydrogels in real–time.

### 5.4. Preparation, Performance, and Mechanism Research of KGM–Based Sensors

**Figure 3:**
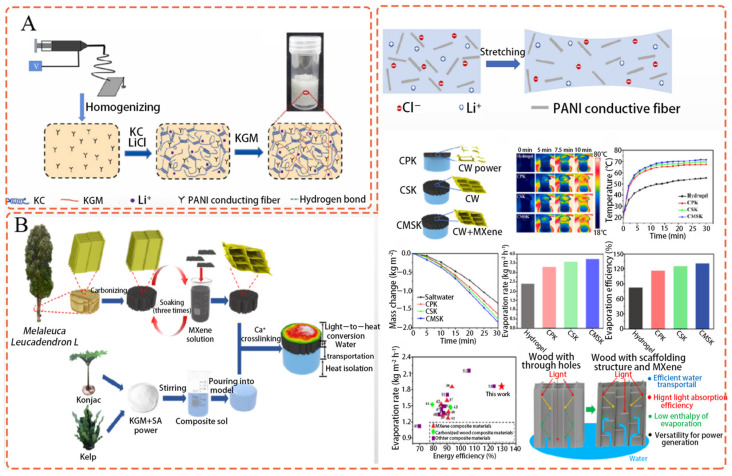
(**A**) Preparation mechanism of conductive hydrogel and schematic illustration of KKLN hydrogel during stretching (Adapted with permission from Ref. [114]. 2023, Elsevier); (**B**) Scheme of the fabrication for the multifunctional evaporator, and the structure, functionality, and working mechanism of the evaporators (Adapted with permission from Ref. [115]. 2023, American Chemical Society Elsevier).

Considerable progress has been made in the field of flexible sensors due to the development of bio–based conductive hydrogels. However, creating bio–based conductive hydrogels with good stability, tensile strength, and electrical conductivity remains challenging. To address this, Chen et al. [114] proposed a straightforward approach for creating conductive, flexible, and stable hydrogels. Figure 3A shows the preparation mechanism of a conductive hydrogel, and a schematic illustration for a KGM/κ–carrageenan/Li/PANI fiber hydrogel (KKLN) hydrogel during stretching. The whole–biological matrix known as KKLN comprised the KGM/κ–carrageenan IPN, which included conducting polyaniline (PANI) fibers that were prepared using the physical form of the lithium ion (Li^+^). The hydrogel exhibited excellent mechanical characteristics (strength: 239.26 kPa, strain: 340.69%) and high electrical conductivity (7261 S/cm) due to the presence of Li^+^ and PANI fiber. Moreover, KKLN exhibited exceptional swelling and heat stability. The produced hydrogel was found to precisely track the motion of body components such as the index finger, elbow, wrist, and knee. The emergence of this preparation method and the advent of electrospinning fiber preparation offer a new approach for developing biomatrix–based flexible sensors.

The development of solar evaporators provides a renewable solution to the energy crisis and lack of fresh water. To create a solar evaporator, Chen et al. [115] used Melaleuca charcoal wood and bio–based hydrogel. The fabrication scheme for the multifunctional evaporator, and the structure, the functionality, and the working mechanism of the evaporators are illustrated in Figure 3B. Multi–layer MXene (Ti_3_C_2_Tx) was included in the scaffold structure of the carbonized wood to create composite carbonized wood. The scaffold structure made of carbonized wood was loose and organized, which significantly increased the capillary force and water transport efficiency. MXene was absorbed by the carbonized wood due to its higher binding energy, lower evaporation enthalpy, and contact angle. MXene also enhanced light absorption, especially for the infrared and ultraviolet wavelengths. By controlling the temperature distribution on the evaporator surface and by concentrating heat, hydrogels made with crosslinking KGM and sodium alginate polysaccharide with Ca^2+^ exhibited low thermal conductivity and enhanced evaporation efficiency. The solar evaporator’s evaporation rate was 3.71 kg/m^2^ h^−1^ under two times the light, the evaporation efficiency was 129.64%, and the open circuit voltage after 20 min of hydropower generation was 1.8 mV, indicating the solar evaporator’s good performance and adaptability. The operation mechanism and design concept of the solar evaporator were understood through experiments and numerical simulations.

## 6. Conclusions

KGM is an important source of raw materials with extensive applications in medical biology due to its unique structure, gelation mechanism, and water retention properties [10,116]. Studies have shown that the biological activity of KGM can be improved through degradation and modification research. The acetyl group plays a crucial role in the atypical helical structure of KGM’s molecular chain, and the degree of polymerization affects the stability of its helical structure. The formation of a topological structure in KGM under external influence is beneficial in improving the stability of active substances.

Hydrogen bonding and hydrophobic interactions establish the three–dimensional gel network in KGM, which closely relates to its hydrophilicity and gel properties. KGM’s biocompatibility, structural compatibility, and excellent properties make it a promising material for tissue engineering scaffolds, wound repair, drug release, endodontic treatments, and 3D printed therapeutic agents. However, the mesoscopic mechanical mechanism of KGM remains unclear, and further research in related areas is necessary. Ongoing research for a non–alkaline–irreversible KGM gel is also promising. 

## Figures and Tables

**Table 2:** KGM as wound dressing material.

Materials	Properties	References
Silk fibroin, KGM	Strong water absorption capacity and similar compressive moist environment with excellent biocompatibility and modulus comparable to skin tissue	[80]
CS, KGM	Biocompatibility with low cytotoxicity and suitable mechanical and barrier properties	[81]
KGM, fish gelatin, matrine	Hemocompatibility and antimicrobial activity	[82]
KGM, human hair proteins, ethanolic extract of Avena sativa	Desirable swelling, biocompatibility, antioxidant activity, and antibacterial activity	[83]
CMKGM, CS	Stable, interconnected pores with high porosity and swelling ability	[84]
KGM, γ–polyglutamic acid	Fast gelation time, little cytotoxicity, good immunomodulation, and antibacterial capabilities with a good water retention rate	[85]
Ace KGM	Hydrophobicity and bioactivity	[43]

## Data Availability

Not applicable.

## References

[1] Ye S., Zongo A.W., Shah B.R., Li J., Li B. (2021). Konjac Glucomannan (KGM), Deacetylated KGM (Da–KGM), and Degraded KGM Derivatives: A Special Focus on Colloidal Nutrition. J. Agric. Food Chem..

[2] Sun J., Jiang H., Wu H., Tong C., Pang J., Wu C. (2020). Multifunctional bionanocomposite films based on konjac glucomannan/chitosan with nano–ZnO and mulberry anthocyanin extract for active food packaging. Food Hydrocoll..

[3] Wang L., Mu R.J., Lin L., Chen X., Lin S., Ye Q., Pang J. (2019). Bioinspired aerogel based on konjac glucomannan and functionalized carbon nanotube for controlled drug release. Int. J. Biol. Macromol..

[4] Pang J., Sun Y.J., Sun Y.M. (2006). Studies on single chain structure of konjac glucomannan. Chin. J. Struct. Chem..

[5] Mu R.-J., Yuan Y., Wang L., Ni Y., Li M., Chen H., Pang J. (2018). Microencapsulation of Lactobacillus acidophilus with konjac glucomannan hydrogel. Food Hydrocoll..

[6] Zhou Y., Jiang R., Perkins W.S., Cheng Y. (2018). Morphology evolution and gelation mechanism of alkali induced konjac glucomannan hydrogel. Food Chem..

[7] Jian W.J., Yao M.N., Wang M., Guan Y.G., Pang J. (2010). Formation Mechanism and Stability Study of Konjac Glucomannan Helical Structure. Chin. J. Struct. Chem..

[8] Lin W., Ni Y., Pang J. (2020). Size effect–inspired fabrication of konjac glucomannan/polycaprolactone fiber films for antibacterial food packaging. Int. J. Biol. Macromol..

[9] Yuan Y., Wang L., Mu R.J., Gong J., Wang Y., Li Y., Ma J., Pang J., Wu C. (2018). Effects of konjac glucomannan on the structure, properties, and drug release characteristics of agarose hydrogels. Carbohydr. Polym..

[10] Zhou N., Zheng S., Xie W., Cao G., Wang L., Pang J. (2021). Konjac glucomannan: A review of structure, physicochemical properties, and wound dressing applications. J. Appl. Polym. Sci..

[11] Ogawa K., Yui T., Mizuno T. (1991). X–ray–Diffraction Study of Glucomannans and Their Acetates. Agric. Biol. Chem..

[12] Jian W.J., Wang M., Yao M.N., Pang J. (2010). Formation Sites and Microscopic Conformation Study on the Konjac Glucomannan Molecular Helices. Chin. J. Struct. Chem..

[13] Jian W.J., Yao M.N., Wan Y., Lin H.M., Zeng Y.A., Pang J. (2011). Study on the Hydration Shell of Single–helical of Konjac Glucomannan. Chin. J. Struct. Chem..

[14] Mao Y.H., Xu Y.X., Li Y.H., Cao J., Song F.L., Zhao D., Zhao Y., Wang Z.M., Yang Y. (2021). Effects of konjac glucomannan with different molecular weights on gut microflora with antibiotic perturbance in in vitro fecal fermentation. Carbohydr. Polym..

[15] Fan L., Peng S., Wen C., He M., Wei X., Wu C., Yao M., Feng R., Pang J. (2012). Analysis of Influential Factors of Konjac Glucomannan (KGM) Molecular Structure on Its Activity. Chin. J. Struct. Chem..

[16] Chen C.Y., Huang Y.C., Yang T.Y., Jian J.Y., Chen W.L., Yang C.H. (2016). Degradation of konjac glucomannan by Thermobifida fusca thermostable beta–mannanase from yeast transformant. Int. J. Biol. Macromol..

[17] Wang S., Zhou B., Wang Y., Li B. (2015). Preparation and characterization of konjac glucomannan microcrystals through acid hydrolysis. Food Res. Int..

[18] Li J., Li B., Geng P., Song A.-X., Wu J.-Y. (2017). Ultrasonic degradation kinetics and rheological profiles of a food polysaccharide (konjac glucomannan) in water. Food Hydrocoll..

[19] Sun Y., Cheng J.Y. (2002). Hydrolysis of lignocellulosic materials for ethanol production: A review. Bioresour. Technol..

[20] Pan T., Peng S., Xu Z., Xiong B., Wen C., Yao M., Pang J. (2013). Synergetic degradation of konjac glucomannan by gamma–ray irradiation and hydrogen peroxide. Carbohydr. Polym..

[21] Hien N.Q., Phu D.V., Duy N.N., Lan N.T.K. (2012). Degradation of chitosan in solution by gamma irradiation in the presence of hydrogen peroxide. Carbohydr. Polym..

[22] Xu Z., Sun Y., Yang Y., Ding J., Pang J. (2007). Effect of γ–irradiation on some physiochemical properties of konjac glucomannan. Carbohydr. Polym..

[23] Jian W., Tu L., Wu L., Xiong H., Pang J., Sun Y.M. (2017). Physicochemical properties and cellular protection against oxidation of degraded Konjac glucomannan prepared by gamma–irradiation. Food Chem..

[24] Morais S., Heyman A., Barak Y., Caspi J., Wilson D.B., Lamed R., Shoseyov O., Bayer E.A. (2010). Enhanced cellulose degradation by nano–complexed enzymes: Synergism between a scaffold–linked exoglucanase and a free endoglucanase. J. Biotechnol..

[25] Jin W., Xu W., Li Z., Li J., Zhou B., Zhang C., Li B. (2014). Degraded konjac glucomannan by γ–ray irradiation assisted with ethanol: Preparation and characterization. Food Hydrocoll..

[26] Wu C., Li Y., Du Y., Wang L., Tong C., Hu Y., Pang J., Yan Z. (2019). Preparation and characterization of konjac glucomannan–based bionanocomposite film for active food packaging. Food Hydrocoll..

[27] Lin W., Ni Y., Wang L., Liu D., Wu C., Pang J. (2019). Physicochemical properties of degraded konjac glucomannan prepared by laser assisted with hydrogen peroxide. Int. J. Biol. Macromol..

[28] Wang L.X., Xiao L.X., Cai L.G., Yin N., Kou D.D., Pang J. (2013). Influence of Konjac Glucomannan on the Crystallization Morphology and Structure of Calcium Oxalate. Chin. J. Struct. Chem..

[29] Pang J., Sun Y., Yang Y., Chen Y., Chen Y., Sun Y. (2008). Studies on hydrogen bonding network structures of konjac glucomannan. Chin. J. Struct. Chem..

[30] Hong X., Ni Y.S., Lin W.M., Mu R.J., Wang L., Pang J., Wu C.H., Wen C.R. (2017). Study on the Epigallocatechin Gallate and Konjac Glucomannan Mosaic Topological Structure. Chin. J. Struct. Chem..

[31] Pang J., Ma Z., Shen B.S., Xu Q.J., Sun Z.Q., Fu L.Q., Fang W.Y., Wen C.R. (2014). Hydrogen Bond Networks’ QSAR and Topological Analysis of Konjac Glucomannan Chains. Chin. J. Struct. Chem..

[32] Chen H., Mu R.J., Pang J., Tan X.D., Lin H.B., Ma Z., Chiang W.Y. (2015). Influence of Topology Structure on the Stability of Konjac Glucomannan Nano Gel Microfibril. Chin. J. Struct. Chem..

[33] Jiang H., Sun J., Li Y., Ma J., Lu Y., Pang J., Wu C. (2020). Preparation and characterization of citric acid crosslinked konjac glucomannan/surface deacetylated chitin nanofibers bionanocomposite film. Int. J. Biol. Macromol..

[34] Yui T., Ogawa K., Sarko A. (1992). Packing Analysis of Carbohydrates and Polysaccharides 18: Molecular and Crystal–Structure of Konjac Glucomannan in the Mannan–II Polymorphic Form. Carbohydr. Res..

[35] Ni Y.S., Mu R.J., Tan X.D., Huang R.X., Yuan Y., Chen H.B., Pang J. (2017). Stability of the Konjac Glucomannan Topological Chain Based on Quantum Spin Model. Chin. J. Struct. Chem..

[36] XiaoYan L., JiuXiang P., LinQiu Y. (2016). Lipase–catalyzed esterification of konjac glucomannan in isooctane. Environ. Prog. Sustain. Energy.

[37] Enomoto-Rogers Y., Ohmomo Y., Iwata T. (2013). Syntheses and characterization of konjac glucomannan acetate and their thermal and mechanical properties. Carbohydr. Polym..

[38] Qiao D., Lu J., Shi W., Li H., Zhang L., Jiang F., Zhang B. (2022). Deacetylation enhances the properties of konjac glucomannan/agar composites. Carbohydr. Polym..

[39] Li Z., Su Y., Xie B., Liu X., Gao X., Wang D. (2015). A novel biocompatible double network hydrogel consisting of konjac glucomannan with high mechanical strength and ability to be freely shaped. J. Mater. Chem. B.

[40] Wang L., Lin L., Pang J. (2020). A novel glucomannan incorporated functionalized carbon nanotube films: Synthesis, characterization and antimicrobial activity. Carbohydr. Polym..

[41] Wei X., Cui C., Fan C., Wu T., Li Y., Zhang X., Wang K., Pang Y., Yao P., Yang J. (2022). Injectable hydrogel based on dodecyl–modified N–carboxyethyl chitosan/oxidized konjac glucomannan effectively prevents bleeding and postoperative adhesions after partial hepatectomy. Int. J. Biol. Macromol..

[42] Xia B., Ha W., Meng X.-W., Govender T., Peng S.-L., Ding L.-S., Li B.-J., Zhang S. (2010). Preparation and characterization of a poly(ethylene glycol) grafted carboxymethyl konjac glucomannan copolymer. Carbohydr. Polym..

[43] Wang C., Li B., Chen T., Mei N., Wang X., Tang S. (2020). Preparation and bioactivity of acetylated konjac glucomannan fibrous membrane and its application for wound dressing. Carbohydr. Polym..

[44] Yu H., Xiao C. (2008). Synthesis and properties of novel hydrogels from oxidized konjac glucomannan crosslinked gelatin for in vitro drug delivery. Carbohydr. Polym..

[45] Wu Z., Tong C., Zhang J., Sun J., Jiang H., Duan M., Wen C., Wu C., Pang J. (2021). Investigation of the structural and physical properties, antioxidant and antimicrobial activity of konjac glucomannan/cellulose nanocrystal bionanocomposite films incorporated with phlorotannin from Sargassum. Int. J. Biol. Macromol..

[46] Xu Z., Yang Y., Jiang Y., Sun Y., Shen Y., Pang J. (2008). Synthesis and characterization of konjac glucomannan–graft–polyacrylamide via gamma–irradiation. Molecules.

[47] Wu X., Xu L.Y., Li E.M., Dong G. (2022). Application of molecular dynamics simulation in biomedicine. Chem. Biol. Drug Des..

[48] Karplus M. (2002). Molecular dynamics simulations of biomolecules. J. Acc. Chem. Res..

[49] Hansson T., Oostenbrink C., van Gunsteren W.F. (2002). Molecular dynamics simulations. Curr. Opin. Struct. Biol..

[50] Hospital A., Goni J.R., Orozco M., Gelpi J.L. (2015). Molecular dynamics simulations: Advances and applications. Adv. Appl. Bioinform. Chem..

[51] Wang L.X., Jiang W., Lin C.P., Zhong Q.X., Pang J. (2014). Studies on the Hydrogen Bonding Network Structures of Amino–konjacglucomannan–zinc Chelate. Chin. J. Struct. Chem..

[52] Wang L.X., Wen C.R., Wu J., Lin H.D., Hu S.G., Pang J. (2013). Studies on the Molecular Chain Conformation Stability of Aminated Konjac Glucomannan. Chin. J. Struct. Chem..

[53] Pang J., Sun Y.J., Guan Y.G., Tian S.P. (2005). Studies on the effect of structure to property stability of glucomannan. Chin. J. Struct. Chem..

[54] Pang J., Sun Y.J., Guan Y.G., Zhu Y.D., Tian S.P. (2005). Molecular dynamics simulation of glucomannan solution. Chin. J. Struct. Chem..

[55] Jiang Y., Reddy C.K., Huang K., Chen L., Xu B. (2019). Hydrocolloidal properties of flaxseed gum/konjac glucomannan compound gel. Int. J. Biol. Macromol..

[56] Li J., Zhang Q., Chang C., Gu L., Su Y., Yang Y., Han Q. (2022). The slow release behavior of soy protein isolate/κ–carrageenan composite hydrogel: Effect of konjac glucomannan. Eur. Polym. J..

[57] Wang M., Yao M.-N., Jian W.-J., Sun Y.-J., Pang J. (2010). Molecular Dynamics Simulations of the Interactions Between Konjac Glucomannan and Soy Protein Isolate. Agric. Sci. China.

[58] Ma Z., Pang J., Lin M.L., Xie B.Q., Chen H., Chen J.L. (2015). Quantum Mechanical Analysis of Sodium Alginate Effects on the Konjac Glucomannan Stability. Chin. J. Struct. Chem..

[59] Roman-Leshkov Y., Barrett C.J., Liu Z.Y., Dumesic J.A. (2007). Production of dimethylfuran for liquid fuels from biomass–derived carbohydrates. Nature.

[60] Huang Y.C., Chu H.W., Huang C.C., Wu W.C., Tsai J.S. (2015). Alkali–treated konjac glucomannan film as a novel wound dressing. Carbohydr. Polym..

[61] Gao S., Guo J., Nishinari K. (2008). Thermoreversible konjac glucomannan gel crosslinked by borax. Carbohydr. Polym..

[62] Song C., Lv Y., Qian K., Chen Y., Qian X. (2019). Preparation of konjac glucomannan–borax hydrogels with good self–healing property and pH–responsive behavior. J. Polym. Res..

[63] Gao S., Guo J., Wu L., Wang S. (2008). Gelation of konjac glucomannan crosslinked by organic borate. Carbohydr. Polym..

[64] Jian W., Zeng Y., Xiong H., Pang J. (2011). Molecular simulation of the complex of konjac glucomannan–borate in water. Carbohydr. Polym..

[65] Hu Y., Tian J., Zou J., Yuan X., Li J., Liang H., Zhan F., Li B. (2019). Partial removal of acetyl groups in konjac glucomannan significantly improved the rheological properties and texture of konjac glucomannan and kappa–carrageenan blends. Int. J. Biol. Macromol..

[66] Wang L.X., Dao L.P., Guo Q.Y., Chen T.L., Fu L.J., Zhou F.C., Yuan Y. (2022). Investigation on the influence of AC electric filed and KCl on the structure and gel properties of Konjac glucomannan. Food Chem..

[67] Oishi K., Maehata Y. (2013). Removal properties of dissolved boron by glucomannan gel. Chemosphere.

[68] Miyoshi E., Takaya T., Williams P.A., Nishinari K. (1996). Effects of sodium chloride and calcium chloride on the interaction between gellan gum and konjac glucomannan. J. Agric. Food Chem..

[69] Filipcsei G., Feher J., Zrinyi M. (2000). Electric field sensitive neutral polymer gels. J. Mol. Struct..

[70] Wei Y., Wang Y.L., He X.J. (2011). Gel Properties of k–Carrageenan–Konjac Gum Mixed Gel and their Influence Factors. Adv. Mater. Res..

[71] Cui B., Chen W., Liang H., Li J., Wu D., Ye S., Li B. (2022). A novel κ–carrageenan/konjac gum thermo–irreversible gel improved by gellan gum and Ca^2+^. LWT.

[72] Wang L., Jiang Y., Lin Y., Pang J., Liu X.Y. (2016). Rheological properties and formation mechanism of DC electric fields induced konjac glucomannan–tungsten gels. Carbohydr. Polym..

[73] Wang L.X., Lee A.R., Yuan Y., Wang X.M., Lu T.J. (2020). Preparation and FTIR, Raman and SEM characterizations of konjac glucomannan–KCl electrogels. Food Chem..

[74] Chen H., Lan G., Ran L., Xiao Y., Yu K., Lu B., Dai F., Wu D., Lu F. (2018). A novel wound dressing based on a Konjac glucomannan/silver nanoparticle composite sponge effectively kills bacteria and accelerates wound healing. Carbohydr. Polym..

[75] Zou Y., Xie R., Hu E., Qian P., Lu B., Lan G., Lu F. (2020). Protein–reduced gold nanoparticles mixed with gentamicin sulfate and loaded into konjac/gelatin sponge heal wounds and kill drug–resistant bacteria. Int. J. Biol. Macromol..

[76] Jiang Y., Huang J., Wu X., Ren Y., Li Z., Ren J. (2020). Controlled release of silver ions from AgNPs using a hydrogel based on konjac glucomannan and chitosan for infected wounds. Int. J. Biol. Macromol..

[77] Liu J., Cui T., Xu X., Du Y., Wang L., Chen S., Pang J. (2022). Robust Alcohol Soluble Polyurethane/Chitosan/Silk Sericin (APU/CS/SS) Nanofiber Scaffolds Toward Artificial Skin Extracellular Matrices via Microfluidic Blow–Spinning. Adv. Fiber Mater..

[78] Zhao X.L. (2013). Antibacterial Heal–Promoting Gel Material Used for Preparing Medical Wound Dressing and Preparation Method Thereof.

[79] Han B.Q., Liu W.S., Peng Y.F., Song F.L., Yang Z. (2013). High–Expansibility Medical Polysaccharide–Based Material and Application Thereof.

[80] Feng Y., Li X., Zhang Q., Yan S., Guo Y., Li M., You R. (2019). Mechanically robust and flexible silk protein/polysaccharide composite sponges for wound dressing. Carbohydr. Polym..

[81] Neto R.J.G., Genevro G.M., Paulo L.A., Lopes P.S., de Moraes M.A., Beppu M.M. (2019). Characterization and in vitro evaluation of chitosan/konjac glucomannan bilayer film as a wound dressing. Carbohydr. Polym..

[82] Zhou L., Xu T., Yan J., Li X., Xie Y., Chen H. (2020). Fabrication and characterization of matrine–loaded konjac glucomannan/fish gelatin composite hydrogel as antimicrobial wound dressing. Food Hydrocoll..

[83] Veerasubramanian P.K., Thangavel P., Kannan R., Chakraborty S., Ramachandran B., Suguna L., Muthuvijayan V. (2018). An investigation of konjac glucomannan–keratin hydrogel scaffold loaded with Avena sativa extracts for diabetic wound healing. Colloids Surf. B Biointerfaces.

[84] Xie Y., Yi Z.X., Wang J.X., Hou T.G., Jiang Q. (2018). Carboxymethyl konjac glucomannan—crosslinked chitosan sponges for wound dressing. Int. J. Biol. Macromol..

[85] Zhu L., Chen J., Mao X., Tang S. (2021). A gamma–PGA/KGM–based injectable hydrogel as immunoactive and antibacterial wound dressing for skin wound repair. Mater. Sci. Eng. C Mater. Biol. Appl..

[86] Zhao Y., Chen H., Ran K., Zhang Y., Pan H., Shangguan J., Tong M., Yang J., Yao Q., Xu H. (2023). Porous hydroxyapatite scaffold orchestrated with bioactive coatings for rapid bone repair. Biomater. Adv..

[87] Kanniyappan H., Thangavel P., Chakraborty S., Arige V., Muthuvijayan V. (2020). Design and evaluation of Konjac glucomannan–based bioactive interpenetrating network (IPN) scaffolds for engineering vascularized bone tissues. Int. J. Biol. Macromol..

[88] Kaisti M., Zenobi-Huang M., Mueller M. (2017). Repair of Cartilage Graft Support and Its Manufacture Method.

[89] Pan X.H., Zhu X.Q., Chen Q., Chen Q.H. (2020). Preparation Method and Application of Cell–Scaffold Composite Material for Cartilage Injury Repair.

[90] Wang J., Chen J., Chen Q.H., Huang M.H. (2012). Hydroxyapatite, Sodium Hyaluronate and Konjac Glucomannan Composite Material and Preparation Method Thereof.

[91] Xu M., Huang J., Jiang S., He J., Wang Z., Qin H., Guan Y.Q. (2022). Glucose sensitive konjac glucomannan/concanavalin A nanoparticles as oral insulin delivery system. Int. J. Biol. Macromol..

[92] Wang C., Guo Z., Liang J., Li N., Song R., Luo L., Ai Y., Li X., Tang S. (2022). An oral delivery vehicle based on konjac glucomannan acetate targeting the colon for inflammatory bowel disease therapy. Front. Bioeng. Biotechnol..

[93] Guerreiro F., Pontes J.F., da Costa A.M.R., Grenha A. (2019). Spray–drying of konjac glucomannan to produce microparticles for an application as antitubercular drug carriers. Powder Technol..

[94] Guerreiro F., Swedrowska M., Patel R., Florez-Fernandez N., Torres M.D., da Costa A.M.R., Forbes B., Grenha A. (2021). Engineering of konjac glucomannan into respirable microparticles for delivery of antitubercular drugs. Int. J. Pharm..

[95] Wang L., Li Y., Lin L., Mu R., Pang J. (2020). Novel synthesis of mussel inspired and Fe(3+) induced pH–sensitive hydrogels: Adhesion, injectable, shapeable, temperature properties, release behavior and rheological characterization. Carbohydr. Polym..

[96] Wang L., Zhou N., Zheng S., Pang J. (2022). Formation of composite hydrogel of carboxymethyl konjac glucomannan/gelatin for sustained release of EGCG. Food Sci. Hum. Wellness.

[97] Dao L., Chen S., Sun X., Pang W., Zhang H., Liao J., Yan J., Pang J. (2022). Construction and sustained release of konjac glucomannan/naringin composite gel spheres. Front. Nutr..

[98] Wu H., Bu N., Chen J., Chen Y., Sun R., Wu C., Pang J. (2022). Construction of Konjac Glucomannan/Oxidized Hyaluronic Acid Hydrogels for Controlled Drug Release. Polymers.

[99] Xie W., Du Y., Yuan S., Pang J. (2021). Dihydromyricetin incorporated active films based on konjac glucomannan and gellan gum. Int. J. Biol. Macromol..

[100] Wu M., Tao B., Wu W. (2022). Anti–washout tricalcium silicate cements modified by konjac glucomannan/calcium formate complex for endodontic applications. Ceram. Int..

[101] Liu C., Xu N., Zong Q., Yu J., Zhang P. (2021). Hydrogel prepared by 3D printing technology and its applications in the medical field. Colloid Interface Sci. Commun..

[102] Wang Y.L., Han L., Zhang X.L., Cao L., Hu K., Li L.H., Wei Y. (2022). 3D bioprinting of an electroactive and self–healing polysaccharide hydrogels. J. Tissue Eng. Regen. Med..

[103] Qin Z., Pang Y., Lu C., Yang Y., Gao M., Zheng L., Zhao J. (2022). Photo–crosslinkable methacrylated konjac glucomannan (KGMMA) hydrogels as a promising bioink for 3D bioprinting. Biomater. Sci..

[104] Wang Y., Han L., Yan J., Hu K., Li L., Zhang H., Ai H. (2019). 3D Bioprintability of Konjac Glucomannan Hydrogel. Mater. Sci..

[105] Ran X., Yang Z., Chen Y., Yang H. (2022). Konjac glucomannan decreases metabolite release of a plant–based fishball analogue during in vitro digestion by affecting amino acid and carbohydrate metabolic pathways. Food Hydrocoll..

[106] Wang Y., Ning Y., Yuan C., Cui B., Liu G., Zhang Z. (2021). The protective mechanism of a debranched corn starch/konjac glucomannan composite against dyslipidemia and gut microbiota in high–fat–diet induced type 2 diabetes. Food Funct..

[107] Zhang W., Li Y., Zhang L., Zhang Q., Liu H. (2022). Preparation of meal replacement powder based on bacterial cellulose/konjac glucomannan and its influence on sugar metabolism. LWT.

[108] Chen H., Nie Q., Hu J., Huang X., Zhang K., Pan S., Nie S. (2019). Hypoglycemic and Hypolipidemic Effects of Glucomannan Extracted from Konjac on Type 2 Diabetic Rats. J. Agric. Food Chem..

[109] Wu D., Yu S., Liang H., Eid M., Li B., Li J., Mao J. (2022). An innovative konjac glucomannan/kappa–carrageenan mixed tensile gel. J. Sci. Food Agric..

[110] Zhang Q., Huang L., Li H., Zhao D., Cao J., Song Y., Liu X. (2022). Mimic Pork Rinds from Plant–Based Gel: The Influence of Sweet Potato Starch and Konjac Glucomannan. Molecules.

[111] Liu L., Zhou N., Yang Y., Huang X., Qiu R., Pang J., Wu S. (2022). Rheological properties of konjac glucomannan composite colloids in strong shear flow affected by mesoscopic structures: Multi–scale simulation and experiment. Colloids Surf. A Physicochem. Eng. Asp..

[112] Xu J., Yang Y., Liu L., Huang X., Wu C., Pang J., Qiu R., Wu S. (2023). Micro–structure and tensile properties of microfluidic spinning konjac glucomannan and sodium alginate composite bio–fibers regulated by shear and elongational flow: Experiment and multi–scale simulation. Int. J. Biol. Macromol..

[113] Zeng X., Yang Y., Liu W., Wen C., Qiu R., Pang J., Wu S. (2022). Real–time quantitative measurement of mechanical properties of spherical hydrogels during degradation by hydrodynamic loading and numerical simulation. Polym. Degrad. Stab..

[114] Chen J., Wang X., Dao L., Liu L., Yang Y., Liu J., Wu S., Cheng Y., Pang J. (2022). A conductive bio–hydrogel with high conductivity and mechanical strength via physical filling of electrospinning polyaniline fibers. Colloids Surf. A Physicochem. Eng. Asp..

[115] Chen J., Jian M., Yang X., Xia X., Pang J., Qiu R., Wu S. (2022). Highly Effective Multifunctional Solar Evaporator with Scaffolding Structured Carbonized Wood and Biohydrogel. ACS Appl. Mater. Interfaces.

[116] Liu Y., Xi Y., Zhao J., Zhao J., Li J., Huang G., Li J., Fang F., Gu L., Wang S. (2019). Preparation of therapeutic–laden konjac hydrogel for tumor combination therapy. Chem. Eng. J..

